# Bevacizumab Addition in Neoadjuvant Treatment Increases the Pathological Complete Response Rates in Patients with HER-2 Negative Breast Cancer Especially Triple Negative Breast Cancer: A Meta-Analysis

**DOI:** 10.1371/journal.pone.0160148

**Published:** 2016-08-31

**Authors:** Xuelei Ma, Xiaoshan Wang, Jingwen Huang, Yingtai Chen, Jing Zhang, Binglan Zhang, Changle Shi, Lei Liu

**Affiliations:** 1 State Key Laboratory of Biotherapy and Cancer Center, West China Hospital, West China Medical School, Sichuan University, Chengdu, Sichuan, China; 2 Department of Clinical Oncology, Sichuan Provincial People’s Hospital, Chengdu, Sichuan, China; 3 Department of Medical Oncology, Cancer Center, West China Hospital, Sichuan University, Chengdu, Sichuan, China; 4 Department of Abdominal Surgery, Cancer Hospital, Chinese Academy of Medical Sciences, Peking Union Medical Collage, Beijing, China; Azienda Ospedaliera di Cremona, ITALY

## Abstract

**Background:**

Neoadjuvant therapy is administered to breast cancer patients as an induction process before surgery or radiotherapy to reduce tumor size. Human epidermal growth factor receptor-2 (HER-2) negative breast cancer lacks effective standard target therapy. Bevacizumab has a controversial role in the treatment of breast cancer and we conduct a meta-analysis to evaluate the value of adding bevacizumab in neoadjuvant regimen.

**Methods:**

Potentially eligible studies were retrieved using PubMed, EMBASE and Medline. Clinical characteristics of patients and statistical data with pathological complete response (pCR) data were collected. Then a meta-analysis model was established to investigate the correlation between administration of bevacizumab in neoadjuvant therapy and pCR rates in HER-2 negative breast cancer.

**Results:**

Seven eligible studies and 5408 patients were yielded. The pCR rates for “breast” or “breast plus lymph node” were similar. In subgroup analysis, we emphasized on patients with triple-negative breast cancer (TNBC). In the criterion of “lesions in breast” the pooled ORs was 1.55 [1.29, 1.86], P<0.00001 and regarding to the evaluation criterion of “lesions in breast and lymph nodes”, the pooled ORs was 1.48 [1.23, 1.78], P<0.0001, in favor of bevacizumab administration.

**Conclusion:**

According to our pooled results, we finally find that bevacizumab addition as a neoadjuvant chemotherapy component, for induction use with limited cycle to improve the pCR rates and patients may avoid long-term adverse event and long-term invalid survival improvement. Especially in subgroup analysis, pCR rates could be improved significantly and physicians could consider bevacizumab with caution. As patients could avoid the adverse event caused by long-term using of bevacizumab, long-term quality of life improvement may be achieved, especially in TNBC.

## Introduction

Breast cancer can be subdivided into human epidermal growth factor receptor 2 (HER-2) positive and HER-2 negative breast cancer due to the important molecular marker HER-2, and around 10%–17% is defined as triple-negative breast cancer (TNBC) that is also negative for estrogen and progesterone receptors It confers a high risk of recurrence and mortality [1]. Neoadjuvant treatment (NT), also called primary systemic treatment, is administered to breast cancer patients as an induction process before surgery or radical radiotherapy to reduce tumor size by allowing for more women to become candidates for breast conserving therapy.

Pathological complete response (pCR) in the breast could be defined as that there is no histologic evidence of invasive tumor foci in the surgical breast specimen (ypT0ypN0/is), while pCR in the breast and axillary nodes was defined as the absence of histologic evidence regarding invasive tumor cells in the surgical breast specimen, axillary nodes identified after neoadjuvant chemotherapy (ypT0ypN0). The pCR rate after NT appears to correlate with improved survival outcome including disease-free (DFS) and overall survival (OS) in local advanced breast cancer patients, and what is more important is that it could act effectively as an indicator for operation [2]. Several recent studies and trials suggested pCR rate used as a surrogate marker for trials comparing different schedules of primary systemic therapy.

Nowadays, choice of treatment for cancer is to combine traditional chemo-radiotherapy with addition of target therapy. For breast cancer, trastuzumab [3, 4] and everolimus [5] were demonstrated to improve the clinical remission rate and survival outcomes in the long-term scale. In a phase III trial, 54.8% HER-2 positive patients receiving trastuzumab plus chemotherapy achieved pCR, but only 19.3% HER-2 positive patients who received chemotherapy alone achieved pCR [6]. The addition of trastuzumab has almost doubled the pCR rates in patients with HER-2 positive breast cancer [6–8].

Meanwhile, bevacizumab, a monoclonal antibody aimed to target vascular endothelial growth factor receptor (VEGFR), still has a controversial role in the treatment of breast cancer. In 2008, Bevacizumab was approved by US Food and Drug Administration (FDA) to treat patients with metastatic breast cancer, which at that time was under an accelerated plan that allows for approval based on data that are not complete enough for the full approval. Later on, the approval of bevacizumab was revoked in 2011 because further studies demonstrated that there was no significant difference regarding overall survival or quality-of-life [9, 10]. Some recent studies demonstrated increased pathological complete response (pCR) rates when adding bevacizumab to the NT in patients with Her-2 negative expression, especially in triple-negative breast cancer (TNBC) type. Bevacizumab was chosen as a candidate choice to further increase the rate of pCR rates in patients with the Her2-negative subtypes.

Previous studies demonstrate that pCR rates ranged from 18% to 61% in various chemotherapy regimens or with different ER/PR expression status. Some conflicting reports still remain in these studies, although patients who received both carboplatin and bevacizumab had the highest pCR rates, the combination did not demonstrate any synergy. The objective of this study was to perform a meta-analysis of recent clinical trials to evaluate the potential value of bevacizumab in NT for patients with HER-2 negative status.

## Materials and Methods

### Search strategy

Pubmed, Medline and EMBASE were searched for the last time on Jun 25, 2016. The search strategy included the following keywords, which are variably combined by “breast cancer”, “neoadjuvant”, “bevacizumab”, and “pathological complete response”.

### Selection criteria

Studies were considered eligible if they met all of the following inclusion criteria, (i) all the patients were local advanced breast cancer patients receiving NT, (ii) receptor expression pattern included were HER-2 negative, (iii) the research investigated the data regarding pCR rate, which is the measurement of the effect of bevacizumab schedule, and (iv) study designs were prospective randomized controlled trials or case control studies. Studies were excluded based on any of the following criteria, (i) were review articles, case report or letters, (ii) lacked key information for calculation pooled ORs from pCR, (iii) single arm studies without control, and (iv) with duplicated data regarding one population.

### Data extraction

All included studies were independently reviewed by two investigators (Huang JW and Ma XL) for data extraction. If there was any discrepancy, we discussed it and further to reach a consensus. The data were independently extracted from eligible studies by two investigators (Huang JW and Ma XL). The primary data were odds ratio (OR) with 95% confidence interval (CI) of pCR after neoadjuvant chemotherapy regimen, with bevacizumab or not.

The additional data obtained from these articles included, first author, publication year, patient source (region), percentage of treatment regimen with bevacizumab, study type, TNM stage, details of neoadjuvant chemotherapy regimens, methods to determine pCR, patients number, who achieved pCR /total patients number in bevacizumab and control group respectively, pCR rates. The statistical data for OR regarding the relationship between bevacizumab administration and pCR rate were also obtained, such as patients number, who achieved pCR /total patients number in bevacizumab and control group respectively.

#### Statistical Methods

The pCR number/total numbers were required in our analysis. The included studies provided the remission and total number of patients in both bevacizumab and control group, and we utilized these primary data to calculate pooled ORs using methods developed by Williamson et al. (2002) [11], and Tierney et al. (2007) [12].

In analysis of pCR rates in patients, the significant outcome was defined as a P value<0.05. A pooled ORs>1 frequently indicated the administration of bevacizumab was related to a relatively better pCR rates. Therefore, we use the term “positive” to describe that bevacizumab administration in neoadjuvant regimen could predicting a better pathological complete response outcome, and “negative” for no correlation between the two neoadjuvant chemotherapy regimens. P<0.10 or I^2^>50% indicates that heterogeneity existing in pooled ORs result (Higgins et al., 2003) [13]. When homogeneity was fine (p≥0.10, I^2^≤50%), a fixed-effects model was applied to secondary analysis; otherwise, a random-effects model was chosen. In terms of publication bias, if the p value greater than 0.10, then the publication bias was accepted in the analysis.

All the earlier calculated ORs were used as measure index to describe the correlation between pCR rates with Bevacizumab adding. The calculation process for the current meta-analysis was performed using REVIEW MANAGER (version 5.0 for Windows; the Cochrane collaboration, Oxford, UK). With regard to publication bias, it was measured using the Begg’s funnel plot, which was performed by STATA 11.0 (STATA Corporation, College Station, TX).

## Result

### Eligible Studies

**Fig 1:**
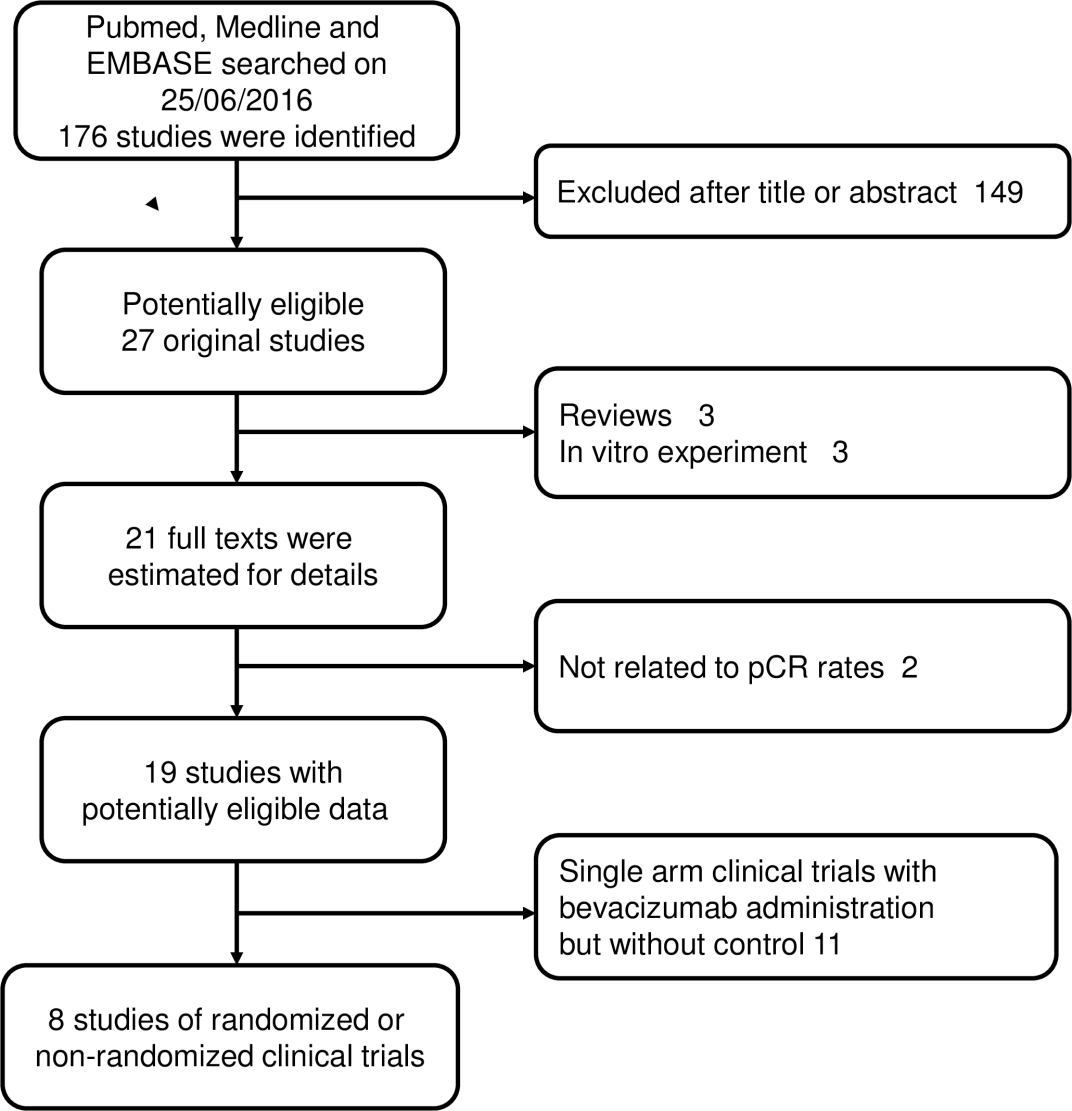
Process of studies selection.

The initial search yielded 176 studies in PubMed, Medline and EMBASE. After a reviewing the abstracts, 27 potentially relevant studies were identified as eligible candidates and underwent a full-text review. Eighteen studies were excluded for the following reasons: three were reviews, eleven were single arm clinical trials without comparison statistics, two were not related to pathologic response rate and three studies were in vitro experiment (Fig 1).

Finally, eight eligible published articles were included [14–20]. In addition, 11 single arm clinical trials containing bevacizumab administration without control has been included as well [16, 21–30]. These eligible control studies were published from 2009 to 2015 and included a total of 5408 patients, ranging from 36 to 1916 per study (median, 703). Five included studies involving eight sets of data related to pooled ORs for pCR rates within breast (ypT0ypN0/is) and five studies with six sets of data dealing with OR data related to pCR rates in the breast and axillary lymph node (ypT0ypN0). The basic clinical characteristics of patients and other useful information were shown in Table 1.

**Table 1:** Basic clinical characteristics of patients.

Author	Diagnostic method	Date	Attitude	Study design	+/- Beva	Age	Type of breast cancer	Receptor statue	Treatment regimen	Stage	pCR define criteria	Beva group		Without beva group		pCR rate
												PCR	total	PCR	total	
Bahri S ^[^^15^^]^	MRI	2009	Negative	Non-RCT	16/20	43	IDC and ILC	HER-2 negative	+/- Beva	Stage II-IV	Breast	5	17	8	20	29.4
Harry D. Bear ^[^^16^^]^	Core biopsy	2012	Positive	RCT	604/602			HER-2 negative	all regimens -AC +/- Beva	cT1—T3,M0	Breast	204	591	168	595	34.5
											Breast & node	167	584	134	591	26.7
									Dox		Breast	63	199	68	201	31.6
									Dox + capecitabine		Breast	74	201	48	204	36.8
									Dox + gemcitabine		Breast	73	204	54	197	35.8
								TNBC			Breast	126	240	116	247	52.5
											Breast & node	105	240	97	247	43.8
								non-TNBC			Breast	84	364	54	355	23.0
Jeon Hor Chen ^[^^17^^]^	MRI and biopsy	2007	Negative	Non-RCT	26/25	48	IBC	HER-2 negative	AC+Taxol or Nab-paclitaxel+Ca+/-beva	Stage II-IV	Breast	1	4	7	12	25
B. Gerber ^[^^18^^]^	Core biopsy	2013	Positive	RCT	323/340	48	Primary IBC	TNBC	EC—Dox +/- Beva		Breast	135	323	104	340	41.8
											Breast & node	127	323	94	340	39.3
Gunter von Minckwitz ^[^^19^^]^	Core biopsy	2014	Positive	RCT	954/962	49	IBC	HER-2 negative			Breast & node	357	956	284	969	18.4
William M. Sikov ^[^^20^^]^	IHC	2014	Positive	Phase II RCT	215/218	40–59	IBC	TNBC	Taxol +/-Beva	Stage II–III	Breast	50	10 5	42	107	47.6
											Breast & node	43	105	39	107	41
									Taxol + Ca +/-Beva		Breast	67	110	53	111	61
											Breast & node	60	110	49	111	54.1
Bernd Gerber ^[^^21^^]^	Core biopsy	2014	Positive	RCT	394/349	48	IDC or IDLC and ILC	HER-2 negative	EC+/-Beva—Dox	cT1-T4	Breast & node	94	394	70	349	23.9
Baljit Singh ^[^^22^^]^	NR	2014	Positive	RCT	173/187	NR	NR	TNBC	+/- Beva	NR	Breast	97	152	73	162	64
											Breast & node	87	152	70	162	57

IHC, immunohistochemistry; RCT, randomized controlled clinical trial; IDC, invasive ductal cancer; ILC, infiltrating lobular cancer; IDLC, invasive ductal-lobular cancer; IBC, invasive breast carcinoma; TNBC, triple negative breast cancer; beva, bevacizumab; +/- beva, with or without bevacizumab; pCR, pathologic complete response; Dox, docetaxel; AC, doxorubicin + cyclophosphamide; EC, etoposide + carboplatin; Nab-paclitaxel, Abraxane-Ab + a new formulation of albumin-bound nanoparticle of paclitaxel; Ca, carboplatin; NR, not reference.

### Correlation between Bevacizumab administration status and pathological complete response

Seven sets of accommodated data showed pathological complete response in patients who were scheduled to receive neoadjuvant chemotherapy plus bevacizumab or neoadjuvant chemotherapy alone. As two different definitions of pCR rates are common: one assessment rule is that no noninvasive residuals could be found in breast (ypT0ypN0/is) and another suggests that no residuals in breast and axillary lymph nodes (ypT0ypN0), we assessed both conditions respectively.

**Fig 2:**
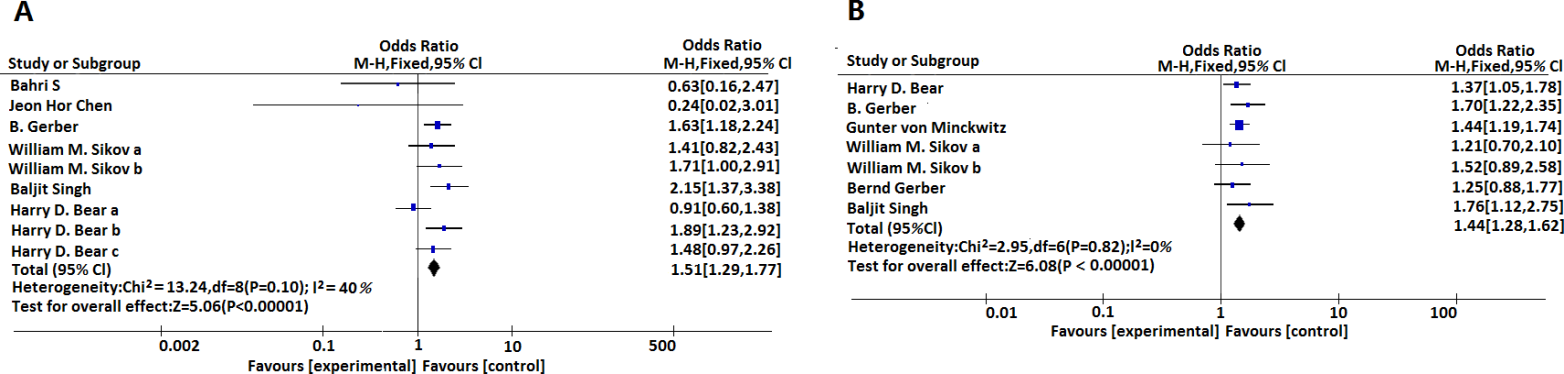
(A) Meta-analysis estimates the relationship between bevacizumab administration and pCR rates (defined as ypT0ypN0/is) in HER-2 negative breast cancer. (B) Meta-analysis estimates the relationship between bevacizumab administration and pCR rates (defined as ypT0ypN0) in HER-2 negative breast cancer. ypT0ypN0/is, absence of histologic evidence of invasive tumor foci in the surgical breast specimen; ypT0ypN0, absence of histologic evidence of invasive tumor cells in the surgical breast specimen and axillary nodes.

After integrating data, we found that pooled ORs to predict the pCR rates for both “breast” and “breast plus lymph node” were similar and the mathematic value for two settings were 1.51 [1.29, 1.77], (I^2^ = 40%, P<0.00001) and 1.44 [1.28, 1.62], (I^2^ = 0%, P<0.00001), respectively. (Fig 2)

### Subgroup analysis

**Fig 3:**
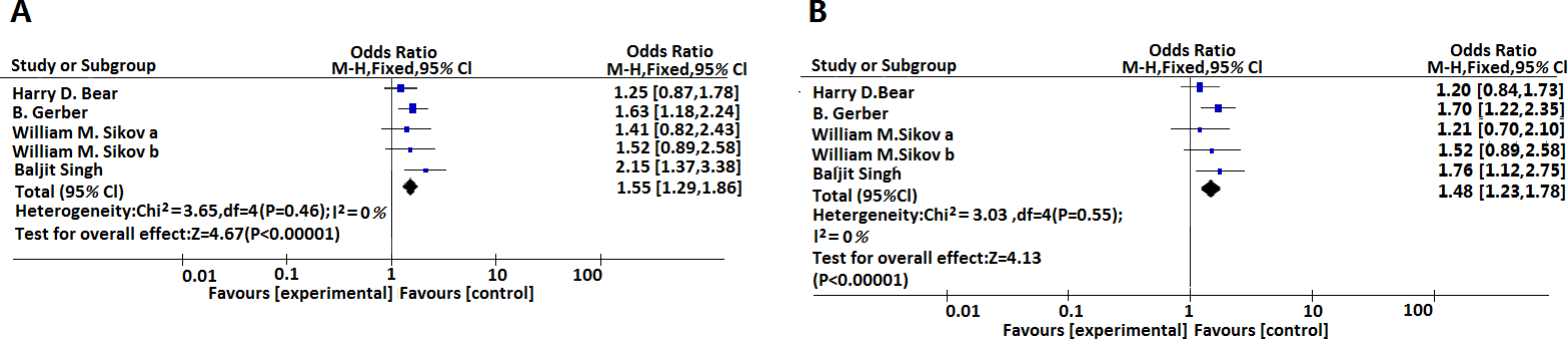
(A) Meta-analysis estimates the relationship between bevacizumab administration and pCR rates (defined as ypT0ypN0/is) in triple negative breast cancer. (B) Meta-analysis estimates the relationship between bevacizumab administration and pCR rates (defined as ypT0ypN0) in triple negative breast cancer. ypT0ypN0/is, absence of histologic evidence of invasive tumor foci in the surgical breast specimen; ypT0ypN0, absence of histologic evidence of invasive tumor cells in the surgical breast specimen and axillary nodes.

In subgroup analysis, we emphasized the patients with triple-negative breast cancer. In the group of “pCR in the breast (ypT0ypN0/is)”, the pooled ORs is 1.55 [1.29, 1.86], (I^2^ = 0%, P<0.00001) and in terms of the evaluation group of “pCR in the breast and axillary nodes (ypT0ypN0/is)”, the pooled ORs was 1.48 [1.23, 1.78], (I^2^ = 0%, P<0.00001). Both standards were effective for prediction patient pCR rates after administration of bevacizumab as a component for neoadjuvant chemotherapy (p<0.05). (Fig 3, Table 2)

**Table 2:** pCR (95% Cl) for evaluation the use of bevacizumab in neoadjuvant treatment.

Receptor status	pCR definition	Study N.	Model	HR (95% Cl)	P value	Heterogeneity (I^2^, p)	Conclusion
HER-2 negative	pCR in the breast	9	Fixed	1.51 [1.29, 1.77]	<0.00001	40%, 0.10	Positive
HER-2 negative	pCR in the breast and axillary nodes	7	Fixed	1.44 [1.28, 1.62]	<0.00001	0%, 0.82	Positive
Triple-negative	pCR in the breast	5	Fixed	1.55 [1.29, 1.86]	<0.00001	0%, 0.46	Positive
Triple-negative	pCR in the breast and axillary nodes	5	Fixed	1.48 [1.23, 1.78]	<0.0001	0%, 0.55	Positive

N, number; HER-2, human epidermal growth factor receptor-2; pCR, pathological complete response; CI, confidence interval.

### Results of single arm studies

Eleven studies aimed to evaluate the remission rate of one group of people with HER-2 negative breast cancers and/or triple-negative subgroup [19, 21–28]. The basic characteristics, including first author, publication year, patient source (region), percentage of treatment regimen with bevacizumab, study type, TNM stage, details of neoadjuvant chemotherapy regimens, methods to determine pCR, patients number who achieved pCR and pCR rate were shown in Table 3. The pCR rates ranged from 9%-37% in the breast (ypT0ypN0/is) and 18%-42% for breast and/or axillary lymph nodes, while the rates were 55% for breast and various from 47%-50% in the breast and/or axillary lymph nodes (ypT0ypN0). (Table 4)

**Table 3:** Basic patients’ characteristics regarding clinical data.

Author	Diagnostic method	Receptor statue	Date	Age	Study design	Stage	Treatment	pCR define cretiria	Beva group	pCR rate (%)	
									pCR	Total	
Mrozek E bu ^[^^23^^]^	Core biopsy	HER-2 negative	2014	48	Single-arm phase II trial	Stage II-III	Nab-P + Ca + beva	Breast and/or axillary lymph nodes	6	33	18%
		TNBC						Breast and/or axillary lymph nodes	6	12	50%
Priya Rastogi ^[^^24^^]^	Core biopsy	HER-2 negative	2011	50	Single-arm phase II trial	Stage IIIA-IIIC	ATC + Cap—beva	Breast	4	45	9%
Sanchez-Rovira ^[^^25^^]^	Core biopsy	HER-2 negative	2013	46	Single-arm phase II trial	Stage IIA-IIIC	AC-Dox + beva	Breast and/or axillary lymph nodes	16	66	24%
Issam Makhoul ^[^^26^^]^	SLNB	HER-2 negative	2013	45	Single-arm phase II trial	Stage IIA-IIIC	ATC + beva	Breast and/or axillary lymph nodes	13	31	42%
		TNBC						Breast and/or axillary lymph nodes	8	14	47%
Clavare-zza M ^[^^27^^]^	Core biopsy	HER-2 negative	2013	48.5	Single-arm phase II trial	Stage IIIA-IIIC	FEC + T + beva	Breast and/or axillary lymph nodes	10	49	21%
		TNBC						Breast and/or axillary lymph nodes	7	15	47%
Jeon Hor Chen ^[^^17^^]^	MRI-guided biopsy	HER-2 negative	2007	51		Stage II-IV	AC + beva	Breast	1	4	25%
Makhoul I ^[^^29^^]^		HER-2 negative	2014	46.5	Single-arm phase II trial	Stage II-III	ATC + beva	Breast	10	27	37%
		TNBC						Breast	6	11	55%
Kim HR ^[^^30^^]^		TNBC	2013	45	Single-arm phase II trial	Stage II-III	Dox + Ca + beva	Breast and/or axillary lymph nodes	8	19	42%
Greil R ^[^^31^^]^	Sentinel node biopsy	HER-2 negative	2009	48	Single-center, phase II	Stage II-III	Dox + Ca + beva	Breast	4	18	22%
Guarneri V ^[59]^	Core-needle biopsy	TNBC	2015	49.4	Single-arm phase II trial	Stage II-IIIC	CaT+beva	Breast and/or axillary lymph nodes	22	44	50%
Bertucci F ^[^^28^^]^	Core biopsy	HER-2 negative	2016	49	Single-arm phase II trial	Stage II-IIIC	FEC + beva	Breast and axillary lymph nodes	19	100	19%

pCR, pathologic complete response; SLNB, sentinel lymph node biopsy; nab-P, nanoparticle albumin-bound paclitaxel; beva, bevacizumab; Ca, carboplatin; FEC, 5-fluorouracil, epirubicin and cyclophosphamide; AC, doxorubicin+ cyclophosphamide; ATC, doxorubicin + docetaxel + cyclophosphamide; ATC+Cap, doxorubicin + docetaxel + cyclophosphamide capecitabine; FEC+T, 5-fluorouracil, epirubicin + cyclophosphamide + Taxol; Dox, dcetaxel; CaT, carboplatin + Paclitaxel.

**Table 4:** pCR (95% Cl) for evaluation the response rate of adding bevacizumab in single-arm study.

Receptor status	pCR definition	Study N.	Response rate range (%)		Mean±SD response rate (%)
HER-2 negative	pCR in the breast	5	9~37	23.3±11.5	
HER-2 negative	pCR in the breast and axillary nodes	4	18~42	26.3±10.8	
Triple-negative	pCR in the breast	1	55	55±0	
Triple-negative	pCR in the breast and axillary nodes	5	42~50	46.5±3.3	

N, number; HER-2, human epidermal growth factor receptor-2; pCR, pathological complete response; CI, confidence interval; SD, standard deviations.

#### Assessment of publication bias

**Fig 4:**
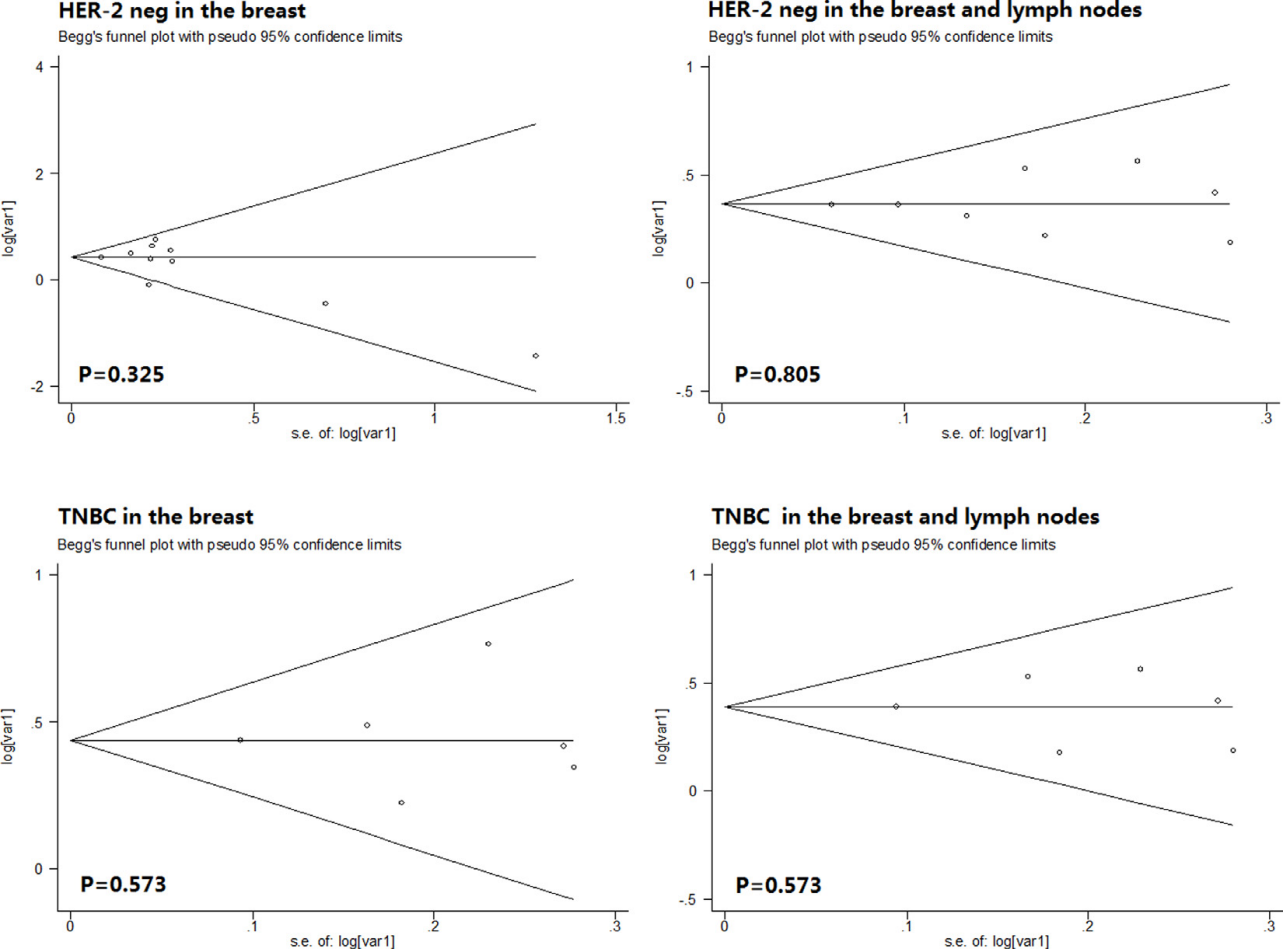
Estimated Begg’s funnel plots of publication bias regarding pCR in the breast of HER-2 negative breast cancer cohort,breast and axillary lymph nodes of HER-2 negative breast cancer cohort, breast of TNBC cohort, breast and axillary lymph nodes of TNBC cohort respectively.

On the basis of Begg’s funnel plot, p value greater than 0.10 indicates that the publication bias was accepted in the analysis. According to the Begg’s funnel plot analysis, publication bias did not emerge in the pCR in the breast of HER-2 negative breast cancer cohort (0.325), pCR in the breast and axillary lymph nodes of HER-2 negative breast cancer cohort (0.805), pCR in the breast of TNBC cohort (0.573) or pCR in the breast and axillary lymph nodes of TNBC cohort (0.573). (Fig 4)

## Discussion

Whether adding bevacizumab into standard neoadjuvant chemotherapy regimen could benefit patients with HER-2 negative breast cancer is controversial, discrepant results regarding bevacizumab addition or not was reported previously. Thus, we conducted a meta-analysis to find out the effect of adding this monoclonal antibody as a target component together with basic chemotherapy. According to our study result, we approved the value of adding bevacizumab into neoadjuvant chemotherapy regimen for patients with HER-2 negative breast cancer, especially people with triple-negative breast cancer (TNBC), which is consistent with previous studies [28, 31, 32].

Marker expression could be used to direct drug administration especially for target therapy, and the utility of target therapy could significantly increase the survival outcomes [33–35] with largely improved quality of life [36, 37]. In breast cancer, HER-2 is a widely used marker for categorizing, as HER-2 positive expression statue is in favor of the usage of trastuzumab [38, 39]. Bevacizumab, a monoclonal antibody proposed to target against vascular endothelial growth factor (VEGF) A and could impair the effect of VEGFR and go against the activated genes of angiogenesis [40, 41]. HER-2 negative breast cancer, especially TNBC are highly invasive type for their high ability of proliferation and enhanced level of VEGFR to prompt angiogenesis [42, 43]. However, as bevacizumab has poor selective characteristics, the toxicity and adverse event are long been discussed. The long-time administration of bevacizumab is difficult for patients to continue, because of the common, high-grade (grade 3 or higher) toxicity and adverse events such as diarrhea, hypertension, and peripheral sensory neuropathy [44].

The dose, time and duration for bevacizumab delivery are three quite important factors influencing the treatment regimen decision. In details, the number of cycle patients received, the utility of bevacizumab in neoadjuvant or adjuvant chemotherapy, and the finish time of it would have considerable effect on the combination with basic chemotherapy and patient remission, survival outcomes. As what was shown from the pooled results, the addition of bevacizumab would improve the pCR rates. Though the CT-NeoBC pooled analysis demonstrated that patients, whoever achieved pathological complete response either in the breast (ypT0ypN0/is) or in the breast and axillary lymph nodes (ypT0ypN0) group had improved survival [45]. It has been demonstrated before that patients, regardless of benefitting from short-term pCR or not, seem to fail to show an inspiring outcomes as no significance has been found regarding the invasive disease-free survival and overall survival [46]. In one recent meta-analysis, the neoadjuvant bevacizumab delivery could improve PFS but not OS regarding to years of survival [47]. However, one previous study reported an opposite result that whether patients achieve pCR after neoadjuvant chemotherapy had a strong positive correlation with surgical rates and survival outcomes (p < 0.05) [48], which give us further clue that patients with pCR after neoadjuvant chemotherapy could increase surgical rate and may further improve the survival outcomes.

While the role of pCR rates to become an independent surrogate marker for predicting survival outcomes is controversial, previous evidence showed established advantage of neoadjuvant chemotherapy (only chemotherapy was administrated) of converting patients who were initially ineligible for breast conserving operations into candidate of this operation on both sides of shrinkage of solid tumor and decrease the incidence of positive nodes [49–51], which is thought to be the first treatment for cancer patients without distant metastasis. Thus, pCR rates after effective neoadjuvant chemotherapy, as in our analysis, the combination of chemotherapy plus bevacizumab, could provide an indication for breast-conserving surgery. In a recent report from National Cancer Database showed that patients who reached pCR, the lumpectomy rate was higher compared to patients who did not achieve pCR (41.0% vs. 26.8%, p < 0.001). In addition, *Rouzier et al* reported that whether patients had complete pCR after neoadjuvant chemotherapy could be an independent predict factor of loco-regional recurrence in patients who underwent breast conserving surgery or mastectomy [52].

In concordance with our result, pCR could be improved in neoadjuvant treatment regimen, which contains bevacizumab and integrating studies described above, we think that pCR is a valuable surrogate marker predicting breast conserving surgery and would further increase quality of life. Thus, based on these clinical trials, short-term results support the adjuvant bevacizumab administration while long-term results go against with the benefit for patients. Thus, for bevacizumab delivery sequence: patients could benefit a lot from neoadjuvant chemotherapy, which plays a role in only 1–2 cycles induction, and after this induction therapy, patients who had better pCR outcomes. Take sever toxicity and patients tolerance into consideration, this short cycle of induction regimen, compared with relative long cycle adjuvant chemotherapy, would be optimal schedule for patients in case of efficiency and toxicity.

In subgroup analysis, patients with TNBC showed better pCR outcomes compared with the whole population. In previous studies, in the result of [NSABP] B-40 trial, patients with TNBC receiving standard chemotherapy plus bevacizumab had significant higher pCR rates compared with no bevacizumab group (P = 0.003). Whereas, GBG44, another randomized controlled study showed no significance between patients with or without bevacizumab (P = 0.43). In accordance with previous studies, our pooled result, with a larger population, indicated that adding of bevacizumab as a component in neoadjuvant chemotherapy would benefit patient with pCR and confer them more opportunities for breast surgery. The pCR is even more important in this subgroup, as data from National Cancer Database showed a strong correlation between improved outcomes in patients with aggressive breast cancer subtypes (TNBC and Her-2 positive breast cancer) and the pCR achievement [15]. One recent meta-analysis of randomized trials regarding bevacizumab plus chemotherapy versus chemotherapy alone to treat non-metastatic breast cancer showed similar trends with ours [53].

In spite that those VEGFR blockers may enhance the severe bleeding after surgery, bevacizumab administration as a neoadjuvant component may be a high risk factor, *Cortés J et al* demonstrated that the surgery could be performed on patients with metastatic breast cancer who underwent bevacizumab therapy before, for the low risk of severe bleeding or wound-healing complications after the surgery [54].

In addition, as this is a meta-analysis, some limitations still exist. Primarily, only published data within those prospective or retrospective studies were included in our meta-analysis, without individual data. In addition, we combined both retrospective and the randomized trials in our meta-analysis and this could also contribute to the bias of this meta-analysis as these two type of studies may not be the same and result in data mixed bias. Also, in pooled data calculation process, we prefer multivariate data if they were available. Otherwise, our calculate data is from the univariate data without adjusting with some other influencing factors, such as age, sex, and Histologic grade. This would bring in a source of bias, for multivariate studies tests the prognostic value independently while univariate studies consider single factor.

Currently, the cost of bevacizumab is a big obstacle preventing its using. Small molecule target therapies designed for breast cancer (including TNBC), such as trastuzumab, are now approved or under clinical trials. Thus, we need further clinical trials to confirm the effectiveness and bright future of bevacizumab. If neoadjuvant regimens contain bevacizumab are really effective and improve pCR or even survival outcomes, the effect overweighs the cost and we could consider bevacizumab.

## Conclusion

In spite of all the limitations and biases of our meta-analysis, we finally find that bevacizumab addition as a neoadjuvant chemotherapy component, for induction use with limited cycle to improve the pCR rates and patients may avoid long-term adverse event and long-term invalid survival improvement. Especially in subgroup analysis, pCR rates could be improved significantly and physicians could consider bevacizumab with caution. As patients could avoid the adverse event caused by long-term using of bevacizumab, long-term quality of life improvement may be achieved, especially in TNBC. Combined with current results, more clinical trials could also focus on the choice of chemotherapy, that is, the effect of bevacizumab plus which chemotherapy could improve pCR rates obviously in a relative cost-effective way.

## Supporting Information

S1 PRISMA Checklist (DOC)
